# First mitochondrial genome of a periwinkle from the genus *Littoraria*: *Littoraria sinensis*

**DOI:** 10.1080/23802359.2019.1692718

**Published:** 2019-11-20

**Authors:** Meng-Yu Li, Yu-Long Li, Teng-Fei Xing, Jin-Xian Liu

**Affiliations:** a CAS Key Laboratory of Marine Ecology and Environmental Sciences, Institute of Oceanology, Chinese Academy of Sciences, Qingdao, China;; b Laboratory for Marine Ecology and Environmental Science, Qingdao National Laboratory for Marine Science and Technology, Qingdao, China;; c Center for Ocean Mega-Science, Chinese Academy of Sciences, Qingdao, China;; d University of Chinese Academy of Sciences, Beijing, China

**Keywords:** Mitochondrial genome, *Littoraria sinensis*, Littorinidae, phylogenetic analysis

## Abstract

The nearly complete sequence of the mitochondrial genome of the periwinkle *Littoraria sinensis* is presented. This is the first mitochondrial genome of the genus *Littoraria* and the fifth sequenced Littorinidae. The mitogenome of *L. sinensis* is 16,320 bp in size, with 37 genes and one partial control region. The genes order is identical to those observed in other Littorinidae mitogenomes. The overall base composition shows that AT content (64.76%) is higher than GC content (35.24%). This study should provide new information for the further phylogenetic studies of this species.

Mitochondrial genome serves as a model for genome evolution and a very powerful tool for revealing evolutionary relationships (Boore 1999). To date, only four mitochondrial genome sequences of the family Littorinidae have been reported (Marques et al. 2017; Fourdrilis et al. 2018). *Littoraria sinensis* belongs to Littorinidae and is one of the most abundant intertidal gastropods along north-western Pacific. However, the identification of different species is still challenging due to morphologically similarity and phenotypic variation in shell morphology (Apolinario et al. 1999; Reid et al. 2010; Lee et al. 2018). Molecular method is very effective for classification, especially for *L. sinensis*, of which the nomenclature has suffered numerous changes (Reid 2001). In this study, we sequenced and annotated the nearly complete mitochondrial genome sequence of *L. sinensis*, which is the first one in the genus *Littoraria*. This study could provide basic information for studying population structure, phylogeographic and phylogenetic relationships in this genus.

The sample of *L. sinensis* was collected from Zhangjiatai, Rizhao, China (35°47′N, 119°61′E) in July, 2018 and mussel tissues were preserved in 90% ethanol. The samples were deposited and stored in Key Laboratory of Marine Ecology & Environmental Sciences, Institute of Oceanology, Chinese Academy of Sciences (KLMEES, IOCAS; 35°58′45″N, 107°42′25″E; Specimen accession number: 2018-ZH-ZJT1). Genomic DNA was extracted following the standard phenol–chloroform extraction protocol and the DNA quality was checked using 1% agarose electrophoresis and Nanodrop 2000c spectrophotometer. Whole genomic shotgun sequencing was performed using the HiSeq X Ten platform (Illumina) at Novogene Co., Ltd. (Beijing, China). Reads with adapter sequences, ambiguous bases (i.e. reads more than 5% unidentified nucleotides) and low-quality sequences (reads with >40% bases having phred quality <15) were removed using fastp (Chen et al. 2018). Then the clean reads were de novo assembled into contig with MEGAHIT (Li et al. 2016). The complete mitogenome sequence of *Littorina saxatilis* was used as a reference to extract candidate mitogenomic contig using Blast+ (Camacho et al. 2009) as implemented in SequenceServer (Priyam et al. 2015). The assembly resulted in an almost complete mitochondrial sequence with a gap in the putative control region. Despite our additional efforts with Sanger sequencing to fill the gap and close the mitogenome, the repetitive content in that were still not fully recovered, which may be filled by long-read sequencing (Marques et al. 2017). The near complete mitogenome of *L. sinensis* was 16,320 bp, with the base composition of 29.79% A, 34.98% T, 20.34% C, and 14.90% G.

The annotation was performed with MITOS WebServer (Bernt et al. 2013). And the results suggested that genes were in the same order with the four reported species of Littorinidae. At last, two ribosomal RNA genes, 13 protein-coding genes, and 22 transfer RNA genes were identified.

The phylogenetic analysis which was constructed using neighbor-joining (NJ) algorithm in MEGA7 (Kumar et al. 2016) showed that *L. sinensis* was phylogenetically divergent to the pervious sequenced genus *Littorina* and *Melarhaphe* (Figure 1). Our results were consistent with the morphological classification research and phylogenetic relationships previously inferred based on several nuclear and mitochondria genes (Williams et al. 2003).

**Figure 1.**
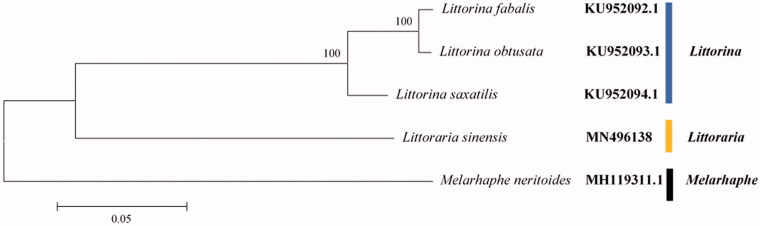
Phylogenetic analyses using NJ algorithm based on the concatenated nucleotide sequences of the 13 PCGs, 22 tRNA and two rRNA genes of 5 species in family Littorinidae. The NCBI accession numbers are indicated after the scientific name and numbers at nodes are bootstrap values.
